# Nestin Is an Independent Predictor of Cancer-Specific Survival after Radical Cystectomy in Patients with Urothelial Carcinoma of the Bladder

**DOI:** 10.1371/journal.pone.0091548

**Published:** 2014-05-02

**Authors:** Ken-ichi Tabata, Kazumasa Matsumoto, Sho Minami, Daisuke Ishii, Morihiro Nishi, Tetsuo Fujita, Makoto Saegusa, Yuichi Sato, Masatsugu Iwamura

**Affiliations:** 1 Department of Urology, Kitasato University School of Medicine, Kanagawa, Japan; 2 Department of Pathology, Kitasato University School of Medicine, Kanagawa, Japan; 3 Department of Applied Tumor Pathology, Kitasato University Graduate School of Medical Sciences, Kanagawa, Japan; Northwestern University Feinberg School of Medicine, United States of America

## Abstract

**Objectives:**

To investigate the association between the expression of nestin, a class VI intermediate filament protein, and pathologic features or survival in patients with urothelial carcinoma of the bladder (UCB).

**Methods:**

Nestin expression in tumor cells was immunohistochemically studied in 93 patients with UCB who underwent radical cystectomy with pelvic lymphadenectomy. The associations with clinicopathologic parameters were evaluated. Kaplan–Meier survival analysis and Cox proportional hazards models were used to estimate the effect of nestin expression on survival.

**Results:**

Nestin expression in cystectomy specimens was observed in 13 of 93 patients (14.0%). Nestin expression was associated with pathologic tumor stage (p = 0.006). Nestin-negative patients had better overall survival compared with nestin-positive patients (log-rank p = 0.0148). Univariable analysis indicated that nestin expression, lymphovascular invasion, and lymph node status were significantly associated with cancer-specific survival (hazard ratios, 2.78, 2.15, and 2.80, respectively). On multivariable analysis, nestin expression and lymph node status were independent prognostic factors in cancer-specific survival (hazard ratios, 2.45 and 2.65, respectively).

**Conclusions:**

The results suggest that nestin expression is a novel independent prognostic indicator for patients with UCB and a potentially useful marker to select patients who may be candidates for adjuvant chemotherapy.

## Introduction

Urothelial carcinoma of the bladder (UCB) is the second most common malignancy and is one of the leading causes of death in patients with genitourinary tract malignancies [1]. Although most patients present with superficial UCB, approximately 50% to 70% of these cases are recurrent; 10% to 15% of these recurrent tumors proceed to both muscle invasion and local extension, which is commonly treated with radical cystectomy [2]. For patients with muscle-invasive and refractory non–muscle-invasive UCB, the standard treatment is radical cystectomy. However, despite advances in surgical techniques and perioperative chemotherapy, the overall 10-year disease-specific survival after radical cystectomy has remained unchanged over recent decades [3], [4]. Although current clinical and pathologic variables provide important prognostic information in bladder cancer, they have limited abilities to predict tumor recurrence or progression and patient survival.

Nestin is a class VI cytoskeletal intermediate filament protein that is expressed in proliferating cells during developmental stages in a variety of embryonic and fetal tissues [5]. In response to injury, the central nervous system, skeletal muscle, and liver can also express nestin [6], [7]. Nestin expression in tumors was originally reported in nervous system malignancies, such as astrocytomas, glioblastomas, and schwannomas [8], [9]. Recently, several studies have reported that nestin is also expressed in other solid carcinomas [10]–[12]. In pancreatic cancer, nestin expression correlates with nerve and stromal invasion [10]. Wolfman et al determined that nestin is a critical component of a novel metastasis pathway in prostate cancer [11]. More recently, Ryuge et al demonstrated that nestin expression is associated with a poorer prognosis and is an independent prognostic factor for survival in patients with non–small-cell lung cancer [12]. The association between nestin expression and clinical outcomes in patients with cancer is gradually being clarified.

In this study, we focused on the relationship between nestin expression levels and clinicopathologic features and survival in patients with UCB after radical cystectomy.

## Materials and Methods

### Patient population

Between March 1990 and November 2006, 133 consecutive patients with UCB who underwent radical cystectomy with pelvic and iliac lymphadenectomy at Kitasato University Hospital were included in this study. Nineteen patients who received preoperative neoadjuvant therapy, including either radiation or chemotherapy, 14 patients who were lost to follow-up, and 7 patients with another type of histology, including adenocarcinoma, squamous cell carcinoma, small-cell carcinoma, and sarcoma, were excluded from further analysis. No patients had distant metastases at the time of cystectomy. Of the remaining 93 patients, the 71 (76.3%) men and 22 (23.7%) women had a median age of 64 years (range, 40–81; mean, 62.5).

The indications for cystectomy in patients with initial Ta, T1, or Tis UCB included intravesical therapy failure (*n* = 14), aggressive histopathologic features (high-grade tumors, carcinoma in situ [CIS], multifocal extensive disease; *n* = 5), or progression to muscle-invasive cancer (*n* = 13). Sixty-one patients underwent cystectomy for an initial presentation of muscle-invasive disease.

The 2002 tumor/node/metastases classification was used for pathologic staging, and the World Health Organization classification was used for pathologic grading. Lymphovascular invasion determined the presence of cancer cells within the endothelial space. Cancer cells that merely invaded a vascular lumen were considered negative. Formalin-fixed, paraffin-embedded blocks representing the most invasive areas of each tumor were collected for further investigation. The median follow-up was 38.4 months (range, 1.1–253.7; mean, 64.6) for patients who were alive at the last follow-up. When patients died, the cause of death was determined by the treating physicians, chart review corroborated by death certificates, or death certificates alone. Autopsy specimens of bladder urothelium from 38 patients without any pathologic findings suggestive of malignancy served as controls.

### Ethical statements

The study protocol was approved by the ethics committee of Kitasato University Hospital (Approved Number; C-09-504). We have obtained written informed consent from all study participants.

### Immunohistochemistry and scoring

Sections (3 µm thick) were deparaffinized in xylene, rehydrated in a descending ethanol series, and then treated with 3% hydrogen peroxide for 10 min. After blocking with 0.5% casein for 10 min, the sections were reacted with 100-times-diluted anti-nestin polyclonal antibody (IBL, Takasaki, Japan) for 2 h at room temperature. The specificity of this antibody was described previously [12]. After rinsing in Tris-buffered saline (TBS; 0.01 M Tris HCl pH 7.5, 150 mM NaCl) 3 times for 5 min each, the sections were reacted with Histofine Simple Stain MAX-PO (MULTI; Nichirei, Tokyo, Japan) for 30 min at room temperature. The sections were visualized subsequently with stable DAB solution (Invitrogen, Carlsbad, CA, USA) and counterstained with Mayer hematoxylin. Batch negative controls used TBS. All slides were reviewed by two investigators (K.M. and Y.S.) who were blinded regarding the clinical and pathologic data. Cytoplasmic immunostaining in tumor cells indicated positivity for nestin. The stainability of peritumoral vascular endothelial cells was used as an internal positive control. Nestin was scored by addition of the percentage of positive tumor cells and staining intensity. The percentage of tumor cells was scored as 0 (0%), 1+ (1−25%), 2+ (26−50%), 3+ (51−75%), and 4+ (75−100%). Staining intensity was also scored as 1+ (weak)  =  weaker than endothelial cells; 2+ (moderate)  =  the same as endothelial cells; 3+ (strong)  =  stronger than endothelial cells. The average of the percentage values obtained by triplicate measurements was considered for analysis. In a preliminary study, we assessed the discriminative value of each cutoff in the sum index, ranging from 0 to 7, with regard to UCB characteristics and prognosis. Kaplan–Meier analyses of serial increments of the sum index as a cutoff for positive and negative revealed that the cutoff of 0 for positive and negative was the best discriminator for UCB progression and survival (data not shown). Likewise, evaluation of the association of this cutoff for nestin with clinical and pathologic characteristics showed that 0 was the only cutoff to yield statistically significant findings (data not shown). Expression scores of nestin were stratified further into positive (score ≥1) and negative (score 0) for presentation.

### Postoperative follow-up

Each patient was scheduled to have a postoperative follow-up every 4 months for the first year, semiannually in the second year, and annually thereafter; more frequent examinations were scheduled if clinically indicated. Twenty-one patients (22.6%) received adjuvant chemotherapy (methotrexate, vinblastine, Adriamycin, and cisplatin [MVAC] or gemcitabine and cisplatin [GC]) after surgery for adverse pathologic characteristics, including regional or distant lymph node metastases or extravesical involvement. Forty-three patients (46.2%) received MVAC or GC for disease recurrence.

### Statistical analyses

For this analysis, tumor pathologic stage (≤pT2 vs. ≥pT3), grade (grades 1 and 2 vs. grade 3), and lymph node status (N0 vs. N1 and N2) were evaluated as dichotomized variables. The Fisher exact test was used to evaluate the association between gender, pathologic stage, pathologic grade, CIS, lymph node status, and lymphovascular invasion. The difference in age between nestin expression and loss groups was tested by the Mann–Whitney *U* test. The Kaplan–Meier method was used to calculate survival functions, and differences were assessed with the log-rank test. Multivariable survival analyses were performed with the Cox proportional hazards regression model, controlling for nestin expression, pathologic stage and grade, concomitant CIS, and lymph node metastases. Statistical significance in this study was set at p<0.05. All reported p values are two-sided. All analyses were performed with Stata version 11 for Windows (Stata, Chicago, IL, USA).

## Results

### Association of nestin expression with clinicopathologic characteristics

**Figure 1:**
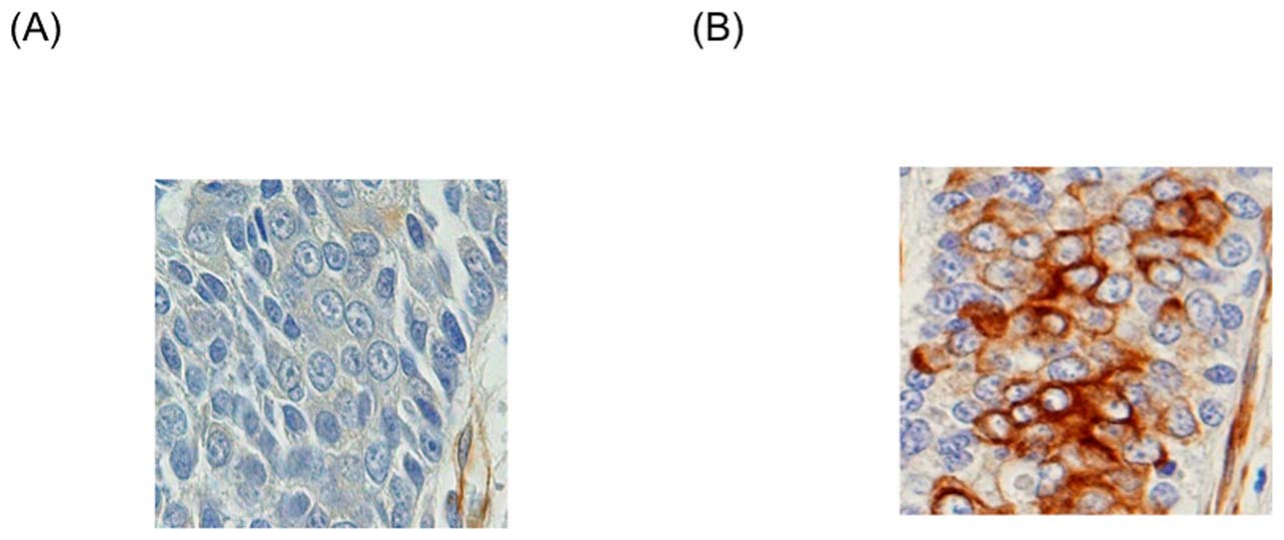
Immunohistochemical analysis of nestin expression in UCB. The photographs are representative tumor cells that were negative (A) and positive (B) for nestin. Nestin expression was also observed in the cytoplasm of vascular endothelial cells and fibroblasts in tumor stroma in each case (Figure 1A, B).

Table 1 summarizes the clinical and pathologic characteristics of 93 patients who underwent radical cystectomy for UCB. Staging indicated that 1 patient had pTis, 2 had pTa, 16 had pT1, 21 had pT2, 17 had pT3a, 19 had pT3b, and 17 had pT4 disease. The correlations between nestin expression and clinicopathologic characteristics are also listed in Table 1. Cytoplasmic nestin expression in tumor cells was observed in 13 of 93 patients (14.0%). The positive rate of nestin was significantly higher in patients with ≥pT3 than in ≤pT2 disease (p = 0.006). Figure 1A demonstrated representative photograph which nestin expression was not observed in tumor cells. Nestin had cytoplasmic expression in tumor cells (Figure 1B). Nestin expression was also observed in the cytoplasm of vascular endothelial cells and fibroblasts in tumor stroma in each case (Figure 1A, B).

**Table 1:** Association of nestin expression with clinical and pathologic characteristics of patients who underwent radical cystectomy for UCB.

		Nestin expression		
	No. of patients	Negative	Positive	p*
Total (%)	93	80(86.0)	13(14.0)	
Gender (%)				
Male	76(81.7)	67(88.2)	9(11.8)	
Female	17(18.3)	13(76.5)	4(23.5)	0.25
pT stage (%)				
≤pT2	40(43.0)	39(97.5)	1(2.5)	
≥pT3	53(57.0)	41(77.4)	12(22.6)	0.006
Pathologic grade (%)				
1 and 2	42(45.2)	38(90.5)	4(9.5)	
3	51(54.8)	42(82.4)	9(17.6)	0.37
Carcinoma in situ (%)				
Negative	81(87.1)	68(84.0)	13(16.0)	
Positive	12(12.9)	12(100)	0(0.0)	0.21
Lymph node status (%)**				
N0	67(72.0)	58(86.6)	9(13.4)	
N1, N2	21(22.6)	19(90.5)	2(9.5)	0.99
Lymphovascular invasion (%)***				
Negative	34(36.6)	32(94.1)	2(5.9)	
Positive	50(53.8)	40(80.0)	10(20.0)	0.11

UCB =  urothelial carcinoma of the bladder; pT =  pathologic tumor.

*Fisher exact test (two-sided).

**Five patients had unknown pathologic status of the lymph nodes.

***Nine patients had no lymphovascular status.

### Relationship between nestin expression and survival in UCB

**Figure 2:**
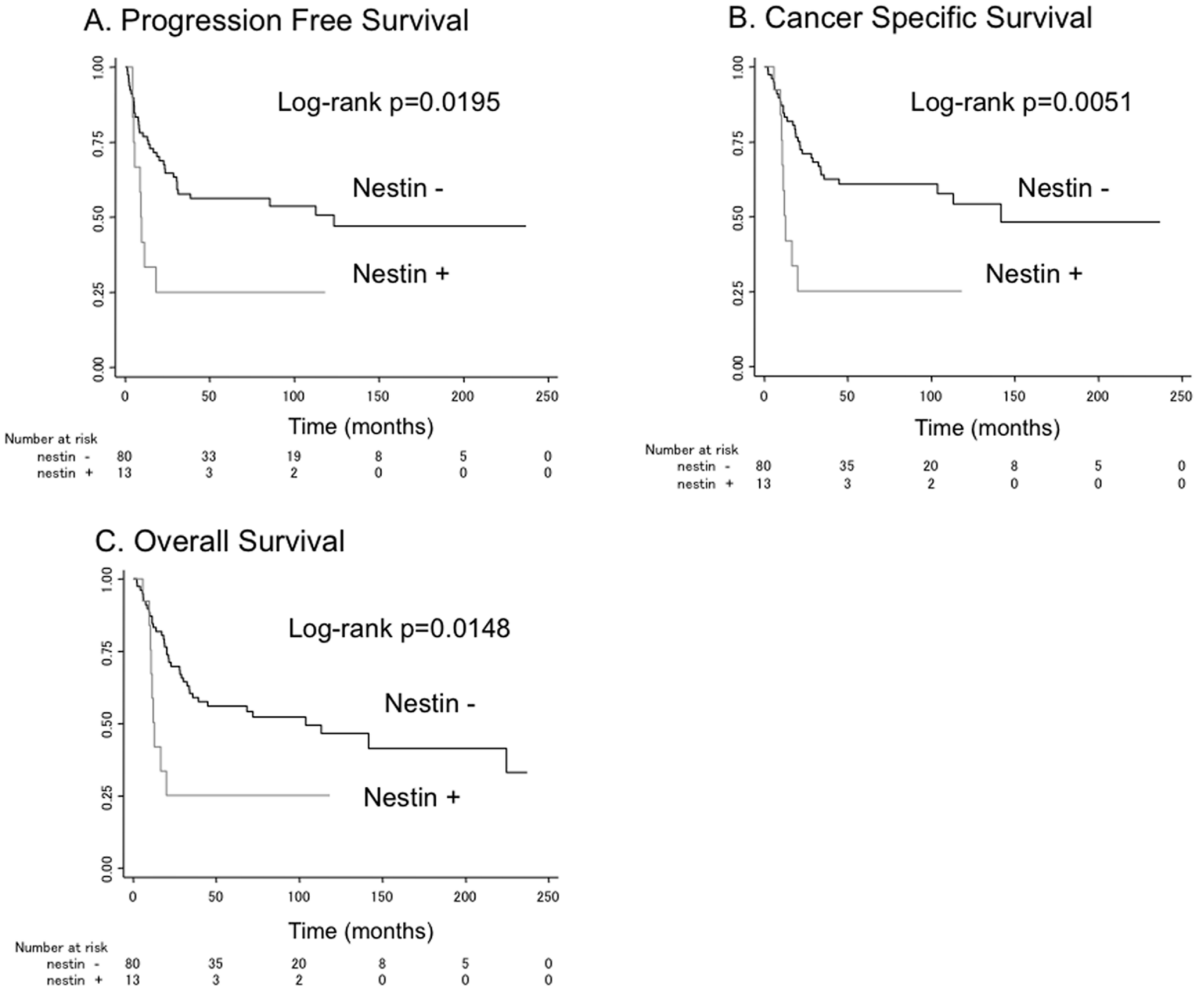
Cumulative survival of UCB patients according to nestin expression estimated sby the Kaplan–Meier method. (A) Progression-free survival; (B) cancer-specific survival; and (C) overall survival.

Within a median follow-up of 38.4 months, 45 patients (48.4%) had disease recurrence, 41 (44.1%) died of UCB, and 7 (7.5%) died of other causes. The association of nestin expression with the rate of progression-free survival and cancer-specific survival and overall survival were analyzed using Kaplan–Meier analysis (Figure 2A–C). The rate of progression-free survival in the nestin-positive group was significantly poorer than that in the nestin-negative group (p = 0.0195, Figure 2A). Significantly poorer rates of cancer-specific survival and overall survival were seen in the nestin-positive group compared with the nestin-negative group (p = 0.0051 and 0.0148, respectively; Figure 2B and C, respectively).

### Prognostic implications of nestin expression in UCB

To investigate whether nestin expression was an independent prognostic factor for survival of patients with UCB, univariable and multivariable Cox regression analyses were used. On univariable Cox regression analysis, nestin expression, pT stage, lymphovascular invasion, and lymph node status were significantly associated with disease recurrence, and nestin expression, lymphovascular invasion, and lymph node status were significantly associated with cancer-specific survival (Table 2). Receiving chemotherapy after cystectomy did not positively affect cancer-specific survival and progression free survival (data not shown). By using the multivariable Cox regression analysis, nestin expression was found to be independent prognostic factor for cancer-specific survival but marginal for disease recurrence (hazard ratio [HR]  = 2.45 and p = 0.04; HR = 2.27; p = 0.06, respectively; Table 2). In this multivariable analysis, lymphovascular invasion was excluded as in a previous report [12] because in determining the effect of nestin on recurrence or survival, lymphovascular invasion might be an intermediate factor in the pathway of the nestin–recurrence/survival relationship.

**Table 2:** Univariable and multivariable analyses for the effect of nestin expression on recurrence and cancer-specific survival.

Factors	HR disease recurrence (p value)*		HR cancer-specific survival (p value)*	
	Univariable	Multivariable	Univariable	Multivariable
Gender				
Gender Male vs. female	0.82(0.61)	1.34(0.55)	0.84(0.67)	0.98(0.96)
Nestin				
Positive vs. negative	2.35(0.02)	2.27(0.06)	2.78(0.008)	2.45(0.04)
pT stage				
≤T2 vs. ≥pT3	2.49(0.04)	1.69(0.27)	2.20(0.08)	1.44(0.45)
Tumor grade				
1, 2 vs. 3	1.73(0.08)	1.45(0.251)	1.58(0.15)	1.24(0.52)
Concomitant CIS				
Positive vs. negative	1.41(0.40)	2.51(0.053)	0.92(0.86)	1.33(0.56)
Lymphovascular invasion				
Positive vs. negative	1.93(0.05)	n/d	2.15(0.029)	n/d
Lymph node status				
N0 vs. N1, 2	3.61(<0.001)	3.59(<0.001)	2.80(0.003)	2.65(0.006)

HR =  hazard ratio; pT =  pathologic tumor; CIS =  carcinoma in situ; n/d =  not done.

*Cox proportional hazard regression.

## Discussion

The prognosis of most patients with advanced UCB is poor even after adequately performed radical cystectomy and pelvic lymphadenectomy [13]. With surgery alone, 40% to 90% of patients with ≥pT3 disease will have distant metastasis or local recurrence or both and will finally die of the disease [4], [13]. Therefore, perioperative additional treatment including systemic chemotherapy should be considered for patients with high risk for disease recurrence if eligible based on the individual's condition. However, to refine clinical decision-making in the selection of additional perioperative treatment for advanced UCB, clinicians need molecular markers associated with the biologic and clinical behavior of these tumors. For example, patients with a low probability of disease progression may be saved the side effects of unnecessary systemic chemotherapy, whereas those with high risk for disease progression can be encouraged to receive systemic chemotherapy. Thus, molecular markers in addition to clinicopathologic features could be help in individualized decision-making with respect to adjuvant chemotherapy.

The present study clarified that nestin expression in tumor cells was associated with progression-free survival, cancer-specific survival, and overall survival of the patients with UCB (Figure 2A–C). Furthermore, in the multivariable Cox regression analysis, nestin expression was an independent prognostic factor for cancer-specific survival (HR, 2.45; p = 0.04; Table 2). Although lymph node status was also an independent prognostic factor for cancer-specific survival (HR, 3.59; p<0.001) and disease recurrence and (HR, 2.65; p = 0.006) in our study, nodal involvement as an independent risk factor after cystectomy for recurrence and survival after cystectomy was already established in several studies [14], [15]. Nestin expression in stromal cells was observed in all cases. We speculated that stromal cells alone do not affect survival or recurrence without nestin positive tumor cells, because stromal cell compartment plays a crucial role in tumorigenesis and invasion by stimulating transformation of normal cells to produce growth factors, cytokines and chemokines that can active tumor cells. In the meantime, lymphovascular invasion was excluded from the multivariable Cox regression analysis in this study. The role of lymphovascular invasion as a prognostic factor for disease progression and survival is controversial. Early studies identified lymphovascular invasion as a poor prognostic features [16], [17]. In other studies, however, lymphovascular invasion was not a predictor of relapse or survival [14], [18], [19]. Previous reports of pancreatic, prostate, and lung cancers revealed that nestin expression was important for migration and invasion capabilities of tumor cells, which subsequently resulted in poor prognoses [10]–[12]. Therefore, lymphovascular invasion was also considered as an intermediate factor in the pathway of the nestin–survival/recurrence relationship in UCB. Previous study also excluded vascular invasion, lymphatic invasion, pleural invasion from the multivariable analysis, and thus, adjusting for them may underestimate the effect of nestin. In addition, patients without lymph node involvement and without nestin expression showed prolonged progression-free survival and cancer-specific survival (data not shown). Therefore, nestin expression possibly helps clinical decision-making in the selection of additional treatment even in patients without lymph node involvement at cystectomy.

On the other hand, epithelial tumors are heterogeneous populations of cells with highly variable abilities to survive, grow, and metastasize [20], [21]. Previous reports suggested that such intratumoral heterogeneity arises from genetic and epigenetic differences of tumor cells through selective pressure during tumor evolution. Recently emerging evidence supports the existence of a cellular hierarchy within epithelial tumors. At the top of this hierarchy is a cancer stem cell (CSC) population that can self-renew and differentiate to progeny cells, thus resulting in the observed cellular and functional heterogeneity of epithelial tumors [22], [23]. CSCs have been isolated from leukemia and several solid tumors [24], [25]. These cells can be expanded in vitro as tumor spheres and are able to reproduce the original tumor when transplanted into immunodeficient mice. The existence of CSCs has also recently been reported in human UCB [26]. Recently, nestin was reported as one of putative markers of CSCs in a variety of tumors including UCB [27], [28]. Previous reports suggested that failure of cancer therapies might be due to their lower effect on CSCs that retain their full capacity to undergo and restore the tumor cell mass. More recently, from this concept, CSC-targeted therapies have been investigated in several tumors [29], [30]. However, the lack of reliable markers for the identification of CSCs prevents the development of this treatment. In this respect, nestin is not only a prognostic factor but also might expand the possibility of the CSC-target treatment in UCB.

This study has limitations inherent to any retrospective data collection. The sample size and variations in follow-up may have limited our ability to detect small differences. However, this study demonstrated that positive nestin expression is associated with poor prognosis and is an independent prognostic factor for cancer-specific survival in patients with UCB after radical cystectomy. In the future, we need a validation of this novel prognostic marker using larger cohort with long-term, prospective study.

## Conclusions

In conclusion, we have demonstrated that nestin is expressed in a subset of UCB and its expression is associated with clinicopathologic factors. We found that nestin expression is a novel prognostic indicator of poor survival for patients with UCB after cystectomy; however, its prognostic significance still requires confirmation with larger patient populations.
